# All‐Cold Evaporation under One Sun with Zero Energy Loss by Using a Heatsink Inspired Solar Evaporator

**DOI:** 10.1002/advs.202002501

**Published:** 2021-02-08

**Authors:** Xuan Wu, Zhiqing Wu, Yida Wang, Ting Gao, Qin Li, Haolan Xu

**Affiliations:** ^1^ Future Industries Institute University of South Australia Mawson Lakes Campus Adelaide South Australia 5095 Australia; ^2^ School of Engineering and Built Environment Griffith University Nathan Queensland 4111 Australia

**Keywords:** heatsink‐like evaporator, high efficiency, photothermal conversion, solar steam generation, zero energy loss

## Abstract

Interfacial solar steam generation is a highly efficient and sustainable technology for clean water production and wastewater treatment. Although great progress has been achieved in improving evaporation rate and energy efficiency, it's still challenging to fully eliminate the energy loss to the surrounding environment during solar steam generation. To achieve this, a novel heatsink‐like evaporator (HSE) is developed herein. During solar evaporation, the temperature on the top solar evaporation surface can be regulated by the fin structures of the HSE. For the evaporators with 5 to 7 heatsink fins, the temperature of the solar evaporation surface is decreased to be lower than the ambient temperature, which fully eliminates the radiation, convection, and conduction heat losses, leading to the absolute cold evaporation over the entire evaporator under 1.0 sun irradiation. As a result, massive energy (4.26 W), which is over 170% of the received light energy, is harvested from the environment due to the temperature deficit, significantly enhancing the energy efficiency of solar steam generation. An extremely high evaporation rate of 4.10 kg m^−2^ h^−1^ is realized with a 6‐fin photothermal HSE, corresponding to an energy conversion efficiency far beyond the theoretical limit, assuming 100% light‐to‐vapor energy conversion.

## Graphical abstract

A heat‐sink like photothermal evaporator is designed and fabricated to realize fully cold solar evaporation under one sun irradiation with zero energy loss and massive energy gain (equivalent to 170% of the incident light energy) from the environment. An extremely high evaporation rate of 4.10 kg m^−2^ h^−1^ with corresponding energy efficiency far beyond the theoretical limit is achieved.

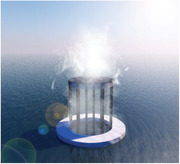

## 1 Introduction

The recently developed interfacial solar steam generation technology offers one of the most promising and cost‐effective ways to produce drinkable water by using clean and renewable sunlight as the energy source.^[^ ^1^, ^2^, ^3^, ^4^, ^5^, ^6^, ^7^, ^8^, ^9^, ^10^, ^11^, ^12^, ^13^, ^14^, ^15^ ^]^ Over the past several years, great efforts have been devoted to structure/configuration design of the solar‐thermal evaporator to increase thermal absorbance, minimize energy loss via radiation, conduction and convection, harvest additional energy from the environment, and recycle latent heat, thereby optimizing solar energy conversion for steam generation.^[^ ^16^, ^17^, ^18^, ^19^, ^20^, ^21^, ^22^, ^23^, ^24^, ^25^, ^26^, ^27^, ^28^, ^29^, ^30^, ^31^, ^32^, ^33^, ^34^, ^35^, ^36^, ^37^, ^38^ ^]^ For instance, in a solar evaporation system where photothermal materials (PTMs) float at water/air interface, the generated heat can be localized at the surface of the PTMs, heating up the thin water film rather than dissipating into bulk water, thus leading to improved energy conversion efficiency.^[^ ^39^, ^40^, ^41^, ^42^, ^43^, ^44^, ^45^ ^]^ In addition, bilayer photothermal structure and photothermal evaporator with spatially separated evaporation surface and bulk water surface were developed, further suppressing the heat conduction loss to the bulk water.^[^ ^12^, ^46^, ^47^, ^48^, ^49^, ^50^, ^51^, ^52^ ^]^ Recently, the heat conduction loss to non‐evaporative bulk water was reported as being phased out by using isolated evaporation structures.^[^ ^53^, ^54^ ^]^ However, in this way part of the generated heat energy was stored within the evaporation system rather than being utilized directly for steam production. On the other hand, the parasitic radiation and convection loss from solar evaporation surface to the surrounding environment could be effectively reduced by decreasing the temperature of evaporation surfaces.^[^ ^25^, ^55^, ^56^, ^57^, ^58^ ^]^ For example, a 3D hollow cone absorber with increased surface area was developed by Zhu's research group, in which the cooling effect caused by the rapid water evaporation led to the reduced surface temperature, therefore contributing to lower radiation and convection losses.^[^ ^56^ ^]^ Distinctly, a 3D cup‐shaped photothermal structure was developed by Wang and co‐workers, in which the energy loss from the evaporation surface was not eliminated, but was recovered by its curved wall structure.^[^ ^57^ ^]^ In the work reported by Gan and co‐workers, cold vapor generation took place by using a 3D triangle structure, where the tilted evaporation surface diluted incident light intensity, resulted in a lower evaporation surface temperature, thus eliminated the thermal radiation and convection loss.^[^ ^55^ ^]^ Moreover, once the temperature of the solar evaporation surface drops below the environment temperature, an extra energy gain from the environment occurs, contributing to extremely high evaporation rates with energy conversion efficiency beyond the theoretical limit, assuming 100% light‐to‐vapor conversion.^[^ ^54^, ^55^, ^59^, ^60^ ^]^ Despite all these progresses, it's still challenging to fully eliminate the energy loss of the evaporation system under one sun irradiation,^[^ ^61^ ^]^ which limits the solar evaporation performance and its practical application under natural sunlight. To address this challenge, herein, a novel 3D heatsink‐like evaporation structure was developed to hit three birds with one stone, which recycles the conduction loss, eliminates the convection and radiation loss, and harvests environmental energy to enhance solar steam generation.

Heatsink is a very common component universally applied in electronic devices. When it is in use, the base surface quickly spreads the generated heat into a cooling medium through its massive fin structures to cool down the heat source in contact with it. Inspired by this, a heatsink‐like photothermal evaporation system was developed, where a top photothermal surface was adopted as the light absorbing platform for light‐to‐heat conversion and solar evaporation. Fin‐like slides were vertically located beneath the top evaporation surface and connected to the bulk water and top evaporation surface. Different from the commercial heatsink where the fins directly dissipate the received heat energy into surrounding medium, here for the heatsink‐like evaporator (HSE), since the surfaces of the fins undergo cold evaporation, the heat transferred from the top evaporation surface is not dissipated into the environment, but is rapidly utilized to enhance water evaporation on the fin surface. By regulating the number of the fin, the temperature of top evaporation surface under 1.0 sun irradiation can drop below the environmental temperature, therefore fully eliminating the radiation and convection losses. By this strategy, it is realized that the temperatures of all the surfaces (top evaporation surface and fin surface) of the HSE are lower than the environment temperature, which induces a massive energy harvest from the surrounding environment, even higher than the incident light energy, significantly enhancing the overall solar evaporation.

To prepare the HSE, bamboo paper with excellent flexibility, mechanical strength, and superhydrophilicity was used to form the frame. For the light absorbing material, carbon materials such as reduced graphene oxide (rGO) and carbon nanotube (CNT) are good candidates due to their excellent light absorption capability, low toxicity, and outstanding physicochemical stability.^[^ ^23^, ^62^, ^63^, ^64^, ^65^, ^66^, ^67^, ^68^, ^69^, ^70^ ^]^ However, the use of rGO and CNTs for practical large scale solar‐steam generation is still facing challenges because of the complicated fabrication procedure along with low production yield, leading to relatively high cost of PTMs. Therefore, it is necessary to develop a facile fabrication procedure to generate carbon materials with comparable light absorbing capacity but low‐cost towards affordable access. Here in this work, porous nanocarbon composites (PCCs) have been produced in high volume by using low cost glycerol as the main carbon source. Moreover, the fabrication process and washing procedure have been greatly shortened and simplified. A light absorption >95% in ultraviolet (UV)‐visible (Vis) regions was achieved over the as‐obtained PCCs, making it ideal for solar‐steam evaporation. By using this HSE design, an extremely high evaporation rate of 4.32 kg m^−2^ h^−1^ was realized under one sun irradiation, which is promising for practical solar steam production.

## 2 Results and Discussion

**Figure 1:**
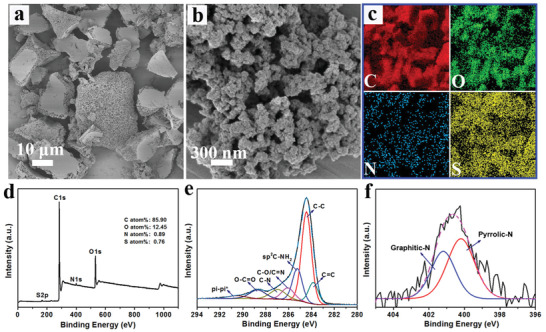
a,b) SEM images and c) element mapping of PCCs. d) XPS survey scan and high‐resolution e) C 1s and f) N 1s spectra of PCCs.

The PCCs were synthesized by a hydrothermal method, in which glycerol was applied as the main carbon source, melamine as the template substrate for the nucleation of the carbon particles. The fabrication process was facile with a high yield of nanocarbon materials. Scanning electron microscopy (SEM) images show that the as obtained carbon material is of porous structure constructed from carbon nanoparticles with a size around 30 nm (**Figure** **1**a,b). The element mapping (Figure 1c) and energy dispersive X‐ray (EDX) spectrum (Figure S1, Supporting Information) reveal that the PCCs are mainly composed of C (>85%). X‐ray photoelectron spectroscopy (XPS) analysis was conducted to further depict the elemental composition and chemical states of the as‐obtained PCCs. As shown in Figure 1d, the XPS survey spectrum of the PCCs shows C 1s peak at 284.5 eV and O 1s at 532.0 eV. The N 1s (400.5 eV) and S 2p (167.5 eV) signals are likely originated from melamine and solvent. The ratio of carbon content was calculated to be 85.9%, which agrees with the EDX report. As shown in Figure 1e, the high resolution C1s peak of the PCCs could be fitted into to C=C (283.9 eV), C—C (284.5 eV), sp^2^ C—NH_2_ (285.3 eV), C—O/C=N (286.1 eV), C—N (287.0 eV), O—C=O (288.8 eV) and *π*—π* shake‐up of the sp^2^ band (290.6 eV). The analysis of N 1s peak shows the existing of sp^3^‐hybridised pyrrolic‐N (400.2 eV) and sp^2^‐hybridised graphitic‐N (401.2 eV) nitrogen bonds (Figure 1f).

**Figure 2:**
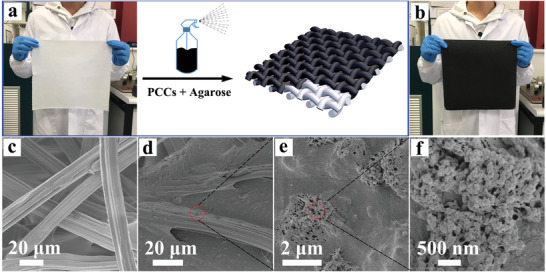
a) Schematic illustration of the preparation of PCCs‐bamboo paper‐agarose PTS. Digital photograph of the a) pristine bamboo paper and b) PTS. SEM images of the c) pristine bamboo paper and d‐f) PTS.

The photothermal aerogel sheet (PTS) was prepared by a facile spray coating method (**Figure** **2**), where a commercial bamboo paper sheet was utilized as the flexible substrate and PCCs as the light absorbing material. The as‐obtained light absorbing materials were first ultrasonicated in a mixture solution (*V*_water_:*V*_ethanol_ = 7:1) to form PCCs substructures (Figure S2, Supporting Information), then mixed with hot agarose solution and sprayed onto the bamboo paper sheet (Figure 2a), which was then pre‐frozen and freeze dried to receive the dark black PCCs‐bamboo paper‐agarose PTS (Figure 2b). As shown in Figure 2c and Figure S3a,b, Supporting Information, the original bamboo paper was simply composed of fiber bundles with a thickness of around 20 µm. After spray coating, the inter gap between the fibers was fully filled with PCCs and agarose (Figure 2d; Figure S3c,d, Supporting Information). High resolution SEM images (Figure 2e,f) clearly demonstrated that the PCCs were embedded and adhered to the surface of bamboo paper fibers by agarose aerogel, which locked the PCCs to avoid the loss of PTM during long‐term application.

**Figure 3:**
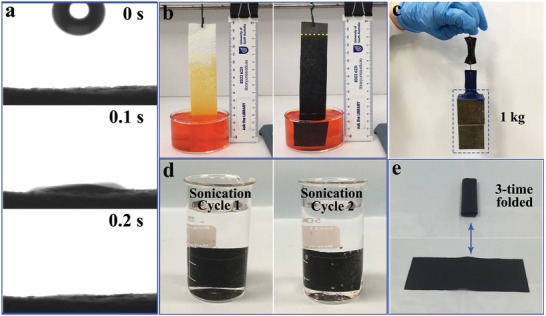
a) Time‐lapse snapshots of absorption of a water droplet by the PTS. b) Vertical water transportation along the pristine bamboo paper (left) and PTS (right) in 5 min, where the dash line indicates the border of water transportation. The concentration of methyl orange solution was 50 mg L^−1^. c) Digital photograph showing the mechanical stability of the wet PST against a pulling weight of 1.0 kg. d) Digital photograph showing the stability of the PTS against two cycles of 5 min sonication, the PTS was kept immersed in water overnight between the two cycles of sonication. e) Photograph illustrating the flexibility of the PTS.

The coating of PCCs and agarose further improved the hydrophilicity of the PTS. As shown in **Figure** **3**a and Video S1, Supporting Information, a water droplet can be absorbed by the PTS within 0.2 s, while for the pristine bamboo paper, it took around 0.3 s (Figure S4 and Video S2, Supporting Information). Consequently, the enhancement in hydrophilicity contributed to the acceleration of water transportation along the PTS. As shown in Figure S5a, Supporting Information, bamboo paper and PTS with the same size (6 × 6 cm) were loaded onto a cuvette with same amount of water over its brim (Figure S5b, Supporting Information). An IR camera was employed to record the water wicking area. Compared with the pristine bamboo paper (Video S3 and Figure S5c–e, Supporting Information), the wicking area of the PTS was about twice large (Video S4 and Figure S5f–h, Supporting Information), confirming better horizontal water transportation ability. Here for the HSE design, the wicking effect of the PTS was again presented vertically to show the water transportation distance against gravity. As confirmed in Figure 3b, the water transportation distance was 10 cm for the PTS and 7.5–8.0 cm for the pristine bamboo paper in 5 min (dash lines in Figure 3b indicating the water transportation height). This result was applied to guide the design of the HSE, in which the length of the photothermal fin was kept at 12 cm and the distance between the top evaporation surface and the bulk water surface was maintained at 8 cm for the continuous water supply. Apart from hydrophilicity, the mechanical stability of the PTS is also important for practical application. Here a pulling strength test showed that a piece of wet PTS (2 cm width × 4 cm length) can easily hold a pulling weight of 1.0 kg (Figure 3c), confirming its excellent mechanical strength. Afterwards, a piece of PTS was sonicated for 5 min and was then kept immersed in water overnight before another 5 min sonication. No black particles were detached from the PTS (Figure 3d), confirming the stable coating of PCCs. The PTS remained intact after being heated in seawater at 50 °C for 8 h (Figure S6, Supporting Information), indicating that it is stable in continuous solar seawater evaporation. In addition, the obtained PTS showed great flexibility, as shown in Figure 3e, a piece of PTS (18 cm in length) could be folded and unfolded many times for easy storage. Due to its excellent mechanical stability and flexibility, the PTS can be readily cut and constructed for 3D evaporation structure design.

**Scheme 1:**
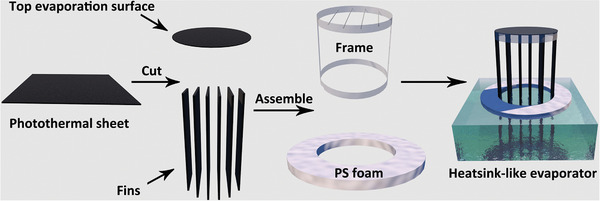
Scheme illustrating the fabrication process of the HSE for solar steam generation.

The fabrication process of the 3D HSE is shown in **Scheme** **1**. The as obtained PTS was first cut into different shapes. The detailed parameters of the PTS for the fabrication of 3D HSE with different fins are shown in Table S1, Supporting Information. The top surface and the fins were assembled by involving a plastic frame as the supporting skeleton (Scheme 1). A piece of polystyrene (PS) foam (1.5 cm in thickness) was applied as the floating insulation layer. The bottom end of the photothermal fins went through the hole of the PS foam for water supply. By controlling the number of fins, a series of PTS‐based HSEs were fabricated (Figure S7, Supporting Information). In addition, a hollow cylinder structure (Figure S7a, Supporting Information) with equivalent top evaporation surface area and same height of side wall was prepared for comparative study.

**Figure 4:**
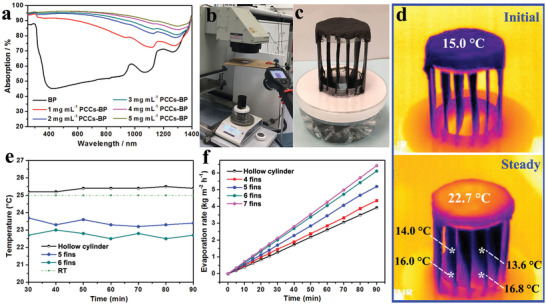
a) Absorption spectra of the pure bamboo paper (wet) and PTSs (wet) with PCCs concentration varied from 1 to 5 mg mL^−1^. b,c) Digital photographs of the test setup for solar steam generation. d) Initial (upper) and steady (lower) IR images of the 6‐fin HSE under 1.0 sun irradiation after 30 min. e) Time‐dependent average temperature of the top evaporation surface with different numbers of fins under 1.0 sun irradiation. f) Time‐dependent weight loss of water over cylinder evaporator and HSEs with different number of fins.

The light absorption of the PTS across the UV‐Vis‐NIR regions (250–1400 nm) was measured prior to solar‐thermal evaporation. As shown in **Figure** **4**a, the light absorption of the PTS generally increased with the increase in concentration of PCCs. For a PCCs concentration of 5 mg mL^−1^, the light absorption of the generated PTS reached >95% in the UV‐Vis range (250–800 nm) and >85% in the NIR region (800–1400 nm). Further increasing the PCCs concentration to 6 mg mL^−1^ could not further improve the light absorption of the PTS (Figure S8, Supporting Information). Therefore, the PTS prepared with a PCCs concentration of 5 mg mL^−1^ was applied for solar‐thermal evaporation. The performance of the HSE was measured under laboratory condition with controlled room temperature (RT: 25 °C) and relative humidity (RH ≈25%). As shown in Figure 4b, a Newport Oriel Solar Simulator (Model: 69 907) was utilized as the light source, and an electronic balance was applied to record the mass change during evaporation. The surface temperature of the photothermal aerogel during evaporation process was monitored by an infrared (IR) camera (FLIR‐E64501). A digital temperature and humidity sensor were utilized for real‐time monitoring of the environment conditions. In addition, since the light spot size was larger than the sample size, an aperture was placed between the sample and the light source to ensure the spot size of the incident light was identical to the size of evaporator, thus avoiding extra energy input into the evaporation system (Figure 4b).^[71]^ An example of the real HSE is shown in Figure 4c. Upon light irradiation, the temperature of the top evaporation surface sharply increased (Figure 4d), indicating the rapid light‐to‐heat conversion. While for the fin‐like structures, the temperature maintained constantly lower than environment temperature (lower image in Figure 4d). For the hollow cylinder evaporation structure, under one sun irradiation, the temperature (average surface temperature) of the top evaporation surface increased from initial 16.9 °C to 22.9 °C within 1 min (Figure S9, Supporting Information). With continued light irradiation, the surface temperature increased gradually and reached a steady evaporation temperature of 25.4 °C after 30 min (Figure 4e; Figures S9 and S10a, Supporting Information), which was slightly higher than the surrounding ambient temperature (25 °C). While for the HSE, the excess heat energy on the top surface could be effectively transferred and consumed by the well‐connected fins for water evaporation. This increased water evaporation drove a certain cooling effect coincided with the remarkable decline of the evaporation temperature, both on the top and fin surfaces (Figure 4e). In addition, it was noticed that the evaporation temperature on the top surface varied with the increase in number of fins. As shown in Figure 4e and Figure S10, Supporting Information, the steady top evaporation surface temperature during solar evaporation was recorded to be 23.3 °C and 22.7 °C for the 5 and 6‐fin evaporators, respectively, both were remarkably lower than the ambient temperature (Figure 4e). Therefore, radiation and convection loss from the top evaporation surface during solar steam generation were fully eliminated. When the fin number was increased to 7, the steady top surface temperature slightly increased to be 23.9 °C (Figures S9 and S10e, Supporting Information), but was still lower than the ambient temperature. This temperature increase might be attributed to the limited space between fins, which led to the accumulation of vapor and high humidity, resulted in decline in evaporation, therefore suppressing energy transfer and consumption from the top surface. In addition, the temperatures of the side wall and fin surfaces of all the evaporators (hollow cylinder and heatsink‐like ones) were all lower than the ambient temperature (Figure S10, Supporting Information). This temperature deficit resulted in a continuous energy harvest from the surrounding environment,^[^ ^55^, ^57^, ^59^ ^]^ contributing to the overall water evaporation of the solar evaporators.

Solar steam generation over the cylinder and HSEs were recorded to evaluate the influence of configuration of the evaporator on the evaporation performance. For the cylinder structure, the steady evaporation rate was 2.65 kg m^−2^ h^−1^, while for the HSEs, the evaporation rate increased with the increase of fin number (Figure 4f), which were 2.89 (4‐fin), 3.48 (5‐fin), 4.10 (6‐fin), and 4.32 (7‐fin) kg m^−2^ h^−1^, respectively. In addition, considering that during solar steam generation, the fins mainly undergo cold/dark evaporation, PTMs are not necessary. Therefore, the photothermal fins were replaced by the pristine bamboo paper fins (Figure S7f, Supporting Information) for comparative study. As shown in Figure S11, Supporting Information, a 10% decrease in evaporation rate (3.66 kg m^−2^ h^−1^) was noticed, which might be ascribed to the insufficient water transportation through the pristine bamboo paper fins, which led to the lower evaporation rate and higher evaporation temperature on the top surface than those of the evaporator with photothermal fins (Figure S10f, Supporting Information).

The light‐to‐vapor energy efficiency (*η*) of the evaporation system was calculated based on the solar energy input and the energy for total vapor output:

(1) $$\eta \;=\frac{m\left(H_{\mathrm{LV}}+Q\right)}{E_{\mathrm{in}}}$$

(2) $$H_{\mathrm{LV}\left(T\right)}=\;1.91846\;\times 10^{6}\;{\left[T_{1}/T_{1}-33.91\right]}^{2}$$

(3) $$Q\;=\;c\left(T_{1}-T_{0}\right)$$

where *m* is the water evaporation rate (kg m^−2^ h^−1^); *H*_LV_ the latent heat required for vaporization of water (kJ kg^−1^); *T*_1_ the temperature of evaporation (K); *Q* the heat for increasing water temperature (J kg^−1^); *c* the specific heat of water (4.2 J g^−1^ K^−1^); *T*_0_ the initial temperature of water; and *E*_in_ (kJ m^−2^ h^−1^) the energy input of the incident light. According to the above equation, assuming 100% of incident light was consumed for solar evaporation, the evaporation rate should be about 1.46–1.47 kg m^−2^ h^−1^. Based on this assumption, the evaporation efficiencies for the HSEs were far beyond the theoretical limit, to be 199.1%, 239.4%, 282.4%, and 297.7% for 4, 5, 6, and 7‐photothermal fin HSEs, respectively. This was due to the additional energy input from the environment rather than incident light. To better understand this extraordinary evaporation efficiency, the energy exchange between the 6‐fin HSE and its surrounding environment was carefully investigated. As shown in Figure 4d,e, during the evaporation process, the temperatures of the top surface (*T*_1_) and the fin surface (*T*_2_) were consecutively lower than the environment temperature *T*_E_, thus convection and radiation energy loss to the environment were fully eliminated. Instead, a massive energy gain from the ambient was driven thoroughly through the entire evaporation surfaces during evaporation. In addition, it is noticed that the bottom end of the fin presented a slightly higher temperature than the top part (Figure 4d; Figure S10d, Supporting Information), indicating an energy flow from the bulk water to the fins.^[^ ^32^ ^]^ Therefore, the energy for solar evaporation (*E*_evap_) could be estimated by using Equation (4):

(4) $$\begin{array}{ccc} & & E_{\mathrm{evap}}=\alpha E_{\mathrm{in}}-A_{1}\varepsilon \sigma \left({T_{1}}^{4}-{T_{E}}^{4}\right)-A_{2}\varepsilon \sigma \left({T_{2}}^{4}-{T_{E}}^{4}\right)\\ & & -A_{1}h\left(T_{1}-T_{E}\right)-A_{2}h\left(T_{2}-T_{E}\right)-Q_{\mathrm{water}} \end{array}$$

where *α* is the optical absorption coefficient; *E*_in_ the input of light energy; *A*_1_ the area of the top surface of the photothermal evaporator (26.40 cm^2^); *T*_1_ the steady average surface temperature of the top surface (22.7 °C); *A*_2_ the surface area of the fin‐like slides; *T*_2_ the average surface temperature of the side wall; *T*_E_ the ambient temperature (25 °C); *ε* emissivity of the absorbing surface (≈0.95); *σ* the Stefan–Boltzmann constant (5.67  ×  10^−8^ W m^−2^ K^−4^); *h* the convection heat transfer coefficient (assumed to be 10 W m^−2^ K^−1^); and *Q*_water_ the energy change between the photothermal evaporator and the bulk water. According to Equation (4), the solar light input *E*_in_ in the 6‐fin heatsink‐like evaporation system was estimated to be about 2.4 W and the radiation and convection energy gain of the top evaporation surface from the environment were estimated to be 0.034 and 0.06 W, respectively. In order to estimate the energy gain of the fins from the surrounding air, a thermocouple was employed to measure the temperature of the air between the fins (Figure S12, Supporting Information). The air temperature between the fins varied between 19.8 °C to 21.4 °C (Figure S12, Supporting Information), which was lower than room temperature but higher than the temperature of the fins. For an easy estimation, the average temperature of 20.6 °C was utilized for calculating the energy gain of the fin from the surrounding medium. While for the fin‐1 and fin‐6 (Figure S13a, Supporting Information), the evaporation on its outside surface happened in open air thus the environment temperature of 25 °C was utilized for the energy calculation. The average surface temperature on each fin was read from IR images (Figure S13b,c, Supporting Information). Due to its symmetrical distribution of the fin structures, the average temperature on fin‐1 and fin‐6 were considered to be the same (15.4 °C), and similar for fin‐2 and fin‐5 (14.6 °C), and fin‐3 and fin‐4 (14.5 °C). Accordingly, the radiation and convection energy harvest of the fins from the environment were estimated to be 1.36 and 2.57 W, respectively. The details of energy harvesting of each fin from the surrounding environment were listed in Table S2, Supporting Information. In addition, to investigate the energy exchange between the 6‐fin HSE and the bulk water, the evaporation system was placed in a thermal insulation Dewar flask (Figure S14a, Supporting Information) with a thermocouple inserted to monitor the temperature change of the bulk water during solar evaporation (Figure S14b, Supporting Information). After 1 h solar evaporation under one sun irradiation, the temperature of the bulk water decreased from the initial 24.9 °C (Figure S14c, Supporting Information) to 24.1 °C (Figure S14d, Supporting Information), indicating a rapid energy transfer from the bulk water to the evaporator for steam generation. According to the equation *Q*_water_ = *cm∆T*, where *c* is the specific heat of water (4.2 Jg^−1^K^−1^), *m* is the weight of bulk water (260 g), and *∆T* is the temperature variation (0.8 K), the energy harvest from the bulk water was calculated to be 0.24 W. Based on the data calculated above, the total energy harvested from the environment medium (air and water) over the 6‐fin HSE during solar evaporation was about 4.26 W, which surprisingly was over 170% of the energy of the incident light (2.4 W), leading to the extraordinary energy efficiency (282.4%) of solar evaporation.

**Figure 5:**
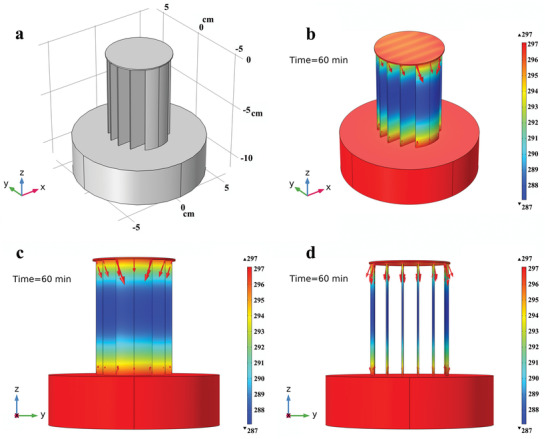
Heat transfer simulation of the 6‐fin HSE during solar steam generation. a) 3D geometry of the HSE with six photothermal fins. b–d) Simulated temperature distribution over the evaporator and the direction of heat flow during solar evaporation. The fins extract heat from both top evaporation surface and bulk water to drive cold evaporation on their surfaces.

The heat flow over the HSE with six photothermal fins was simulated by using numerical simulation software (COMSOL 5.5). A 3D geometry with the exact same dimensions of the HSE was first constructed (**Figure** **5**a). The temperature distribution over the evaporation system was simulated by the heat transfer module (See Experimental Section). The simulation results showed the temperature distribution and heat flow over the evaporation system (Figure 5). The fins, which connected the bulk water and the top evaporation surface, presented the lowest temperature (Figure 5b,c). Due to the temperature gradients, the fins extracted conductive heat from both the top evaporation surface and the beneath bulk water as shown by the red arrows whose sizes were proportional to the magnitude of heat flux (Figure 5b–d). This energy transfer caused the decrease of the temperature of the top evaporation surface and the bulk water, which agreed well with the experimental results.

**Figure 6:**
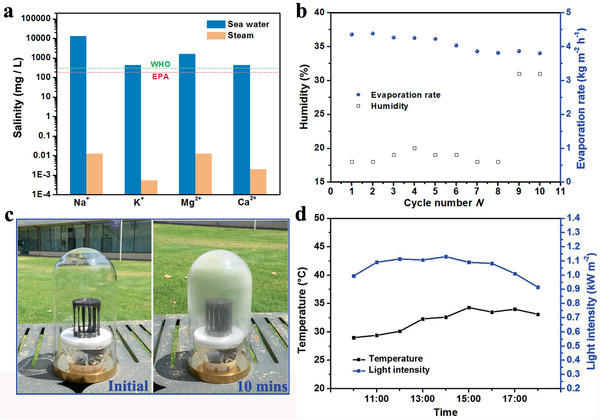
a) Ion concentrations of the original seawater and the condensed steam collected during solar‐thermal evaporation. b) Cycle performance of HSE with 6‐photothermal fin under 1.0 sun irradiation for sea water evaporation. Digital photographs showing the c, left) water collection device for the solar‐thermal evaporation and c, right) the condensation of clean water on the shell. d) Light intensity and temperature during the outdoor testing.

Real seawater (Semaphore Beach, Adelaide, South Australia) evaporation under one sun irradiation was performed to further explore the feasibility of the HSE for desalination. The salinity of the collected clean water from the solar evaporation of the 6‐fin HSE was measured by inductively coupled plasma‐mass spectrometry (ICP‐MS). As shown in **Figure** **6**a, the concentration of all four major ions (K^+^: 0.53 ppb, Ca^2+^: 2.0 ppb, Na^+^: 12.56 ppb, and Mg^2+^: 12.50 ppb) in the collected water were much lower than the salinity level determined by both the World Health Organization (WHO) and US Environmental Protection Agency (USEPA) Standard for drinkable desalinated water (https://www.who.int/water_sanitation_health/publications/dwq-guidelines-4/en/). The seawater evaporation performance of the HSE (6‐photothermal fin) was recorded for 10 cycles over two days under 1.0 sun irradiation (Figure 6b). The evaporation rates were stable in the range of 3.80–4.39 kg m^−2^ h^−1^. The slight fluctuation in solar evaporation rate was due to the variation in the environmental humidity.^[^ ^55^, ^72^ ^]^

The water collection performance test over this HSE under natural sunlight irradiation was carried out in a glass dome (with a hole on the base as clean water outlet) from 10 am to 6 pm (Figure 6c). The outdoor tests were carried out at Mawson Lakes Campus (34.9229° S, 138.5912° E), University of South Australia, Adelaide, Australia on 25^th^ December 2019. Upon sunlight irradiation, water vapor was quickly generated, and a thin condensed water film immediately formed on the inner wall of the glass dome (Figure 6c). The weather conditions during the evaporation test was recorded and shown in Figure 6d. During the outdoor test, 77.78 g clean water over 8 h evaporation was generated, corresponding to an average evaporation rate of 3.68 kg m^−2^ h^−1^. Outdoor seawater desalination under natural sunlight was also carried out on three different days (8 h per day). The weather conditions, light intensities, environmental temperatures, and average evaporation rates were shown in Figure S15 and Table S3, Supporting Information. It was noticed that after 8 h continuous outdoor seawater evaporation, no salt crystal was formed and accumulated on the surface of the HSE (Figure S16, Supporting Information), indicating that this evaporator is applicable for practical seawater desalination.

## 3 Conclusion

In this work, a novel HSE was developed, showing for the first time that a photothermal evaporator completely eliminated the convection and radiation losses under one sun radiation. Moreover, the evaporator was able to recycle the conductive heat loss, as well as to harvest energy from the environment and bulk water, contributing to extremely high energy efficiency of solar steam generation. The HSE was comprised of a top circular surface connected with fin structures, which were made of bamboo paper coated with cost‐effective porous carbon material. Upon light irradiation, the top surface quickly converted the received light energy into heat to drive the solar evaporation. Meanwhile the excess heat energy was injected into the attached fins to facilitate the cold evaporation thereof, leading to the decline in the temperature of the top evaporation surface. By increasing the number of fins, the temperature of the top evaporation surface decreased to be lower than the environment temperature during solar evaporation under one sun irradiation, thus fully eliminating the radiation and convection heat losses. On the other hand, the conduction heat energy transferred from the top surface was recycled and consequently consumed by the cold water evaporation on the fin slides. Benefitting from this structure design, the evaporation temperature of the whole device was consecutively maintained below ambient temperature, which for the first time realized full cold evaporation under one sun irradiation. In addition, a massive energy equivalent to 170% of the incident light energy was harvested from the surrounding environment, significantly improved the evaporation rate (4.10 kg m^−2^ h^−1^, 6 fin HSE) with corresponding energy efficiency of 282.4%, assuming 100% light‐to‐vapor energy conversion. Seawater evaporation under one sun irradiation was conducted to evaluate the feasibility of the HSE for desalination. A Na^+^ concentration of 12.56 ppb was measured in the collected steam, which was much lower than the salinity level marked by WHO and USEPA for drinkable desalinated water. Therefore, this HSE with outstanding energy efficiency showed great potential for real‐world applications for clean water production.

## 4 Experimental Section

##### Materials and Chemicals

Bamboo paper was purchased from the local market. Glycerol, melamine, and agarose was purchased from Sigma‐Aldrich. Sulfuric acid (98%) was purchased from Alfa Aesar. Urea and ethanol were purchased from Chem‐Supply. Unless otherwise noted, Milli‐Q water with a resistance >18.2 MΩ cm^−1^ was used for all experiments.

##### Preparation of PCCs

In a 50 mL Teflon‐lined stainless‐steel autoclave, melamine (0.5 g) was added to 10 mL of glycerol and stirred till melamine was fully dissolved. Afterwards, 10 mL 98% sulfuric acid was added into the above mixture under vigorous stirring. The autoclave was sealed and heated at 180 °C for 4 h, and then cooled down naturally till ambient temperature. The resulting mixture was washed and centrifuged with water and ethanol until neutral. The obtained PCCs were then redispersed with ultrasonication (QSonica, model: Q125) in a mixture solution of Milli‐Q water and ethanol (*V*_water_: *V*_ethanol_ = 7:1) to generate a homogenous suspension.

##### Preparation of PCCs‐Bamboo Paper‐Agarose Aerogel Sheet

Urea (0.125 g mL^−1^) and agarose (12.5 mg mL^−1^) were added into PCCs dispersion (80 mL of 1–5 mg mL^−1^), which was stirred at 80 °C for 30 min to obtain a homogenous black PCCs‐agarose suspension. Afterward, the produced black hot dispersion was spray coated onto bamboo paper sheet (28 cm × 30 cm), followed by prefrozen and freeze drying.

##### Characterization

SEM images were obtained on a Zeiss Merlin SEM. XPS analysis was carried out on a Kratos Axis Ultra with a Delay Line Detector photoelectron spectrometer using an aluminum monochromatic X‐ray source. UV‐Vis spectra were recorded using a UV‐2600 Spectrophotometer (Shimadzu). Infrared photographs were captured using an IR camera (FLIRE64501). A DataPhysics OCA 20 contact angle system was employed to characterize the hydrophilicity of the samples. Initial concentrations of common cations present in seawater (from Semaphore Beach, Adelaide, Australia) were measured using an Inductively Couple Plasma Optical Emission Spectrometry (ICP‐OES, Optima 5300 V, Perkin Elmer). Following desalination trace levels of residual ion concentrations in the collected clean water were analyzed using an Inductively Couple Plasma‐Mass Spectrometry (ICP‐MS) Triple Quad system (ICP‐QQQ, Agilent 8800).

##### Solar‐Driven Steam Generation

Solar‐driven steam generation was recorded under laboratory conditions (ambient temperature: 25 °C). A Newport Oriel Solar Simulator (Model: 69 907) was used as the light source, and an electronic balance was connected to record the mass change during evaporation. The surface temperature of the photothermal aerogel was monitored by an infrared (IR) camera. A digital temperature and humidity sensor was applied for the real time monitoring of the evaporation environment. In addition, an aperture with a hole size matching the size of the photothermal evaporator was applied to block any extra irradiation from the light source.

##### Heat Flow Simulation by Commercial COMSOL Multiphysics 5.5

The temperature distribution and heat flow over the evaporation system were simulated by the heat transfer module, where the bamboo paper‐based evaporator was regarded as a porous medium (PM). The following equations were used for the steady‐state heat transfer modeling in a porous matrix filled with a fluid:

(5) $$Q\;={\left(\rho C_{p}\right)}_{\mathrm{eff}}\;+\rho C_{p}u\cdot \nabla T+\nabla \cdot q\;$$

(6) $$q\;=\;-k_{\mathrm{eff}}\nabla T$$

(7) $${\left(\rho C_{p}\right)}_{\mathrm{eff}}=\theta_{p}\;\rho_{p}C_{p,p}+\left(1-\theta_{p}\right)\rho C_{p}$$

(8) $$k_{\mathrm{eff}}=\theta_{p}\;k_{p}+\left(1-\theta_{p}\right)k$$

where *Q* (W m^−3^) is the heat flux; *ρ* (1000 kg m^−3^) is the fluid density; *C*_p_ (4200 J kg^−1^ K^−1^) is the fluid heat capacity at constant pressure; (*ρC*_p_)_eff_ is the effective volumetric heat capacity at constant pressure; *u* is the Darcy velocity (the volume flow rate per unit cross sectional area, m s^−1^); the velocity within the pores can be calculated as *u*_L_ = *u*/*θ*_p,_ where *θ*_p_ (80%) represents the porosity of the material; ∇*T* stands for the temperature gradient (K m^−1^); *q* is the heat flux vector (W m^−2^); *k*_p_ (0.02 W m^−2^ K^−1^) and *k*_eff_ stand for the thermal conductivity and effective thermal conductivity (W m^−1^ K^−1^) of the porous matrix, respectively.

## Conflict of Interest

The authors declare no conflict of interest.

## Supporting information

Supporting Information

Supplemental Video 1

Supplemental Video 2

Supplemental Video 3

Supplemental Video 4
