# Supplementary material for: All‐Cold Evaporation under One Sun with Zero Energy Loss by Using a Heatsink Inspired Solar Evaporator
Source: Adv Sci (Weinh). 2021 Feb 8;8(7):2002501. doi: 10.1002/advs.202002501 (PMC8025000; doi:10.1002/advs.202002501)
Supplement: Supplementary file 1 — Supporting Information [file ADVS-8-2002501-s003.pdf]

WILEY-VCH

© 2021 Wiley-VCH GmbH

ADVANCED SCIENCE
Open Access

Supporting Information

for *Adv. Sci.*, DOI: 10.1002/advs.202002501

All-Cold Evaporation under One Sun with Zero Energy Loss by Using A Heatsink Inspired Solar Evaporator

Xuan Wu,[1†] Zhiqing Wu,[2†] Yida Wang,[1†] Ting Gao,[1] Qin Li,[2] Haolan Xu[1*]*

© 2020 Wiley-VCH GmbH

Supporting Information

All-Cold Evaporation under One Sun with Zero Energy Loss by Using A Heatsink Inspired Solar Evaporator

Xuan Wu,[1†] Zhiqing Wu,[2†] Yida Wang,[1†] Ting Gao,[1] Qin Li,[2] Haolan Xu[1*]*

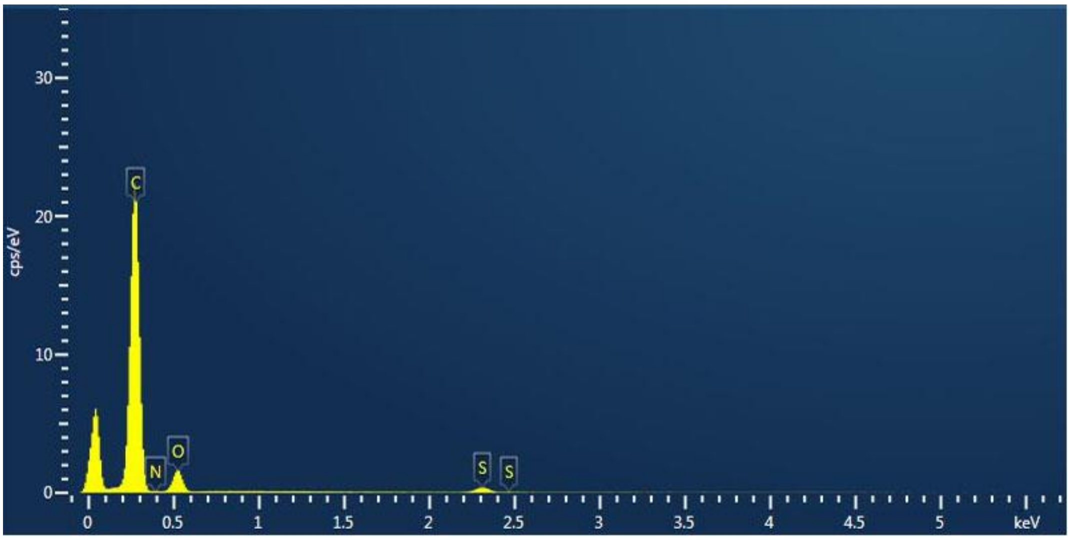

Figure S1. EDX spectrum of the generated PCCs.

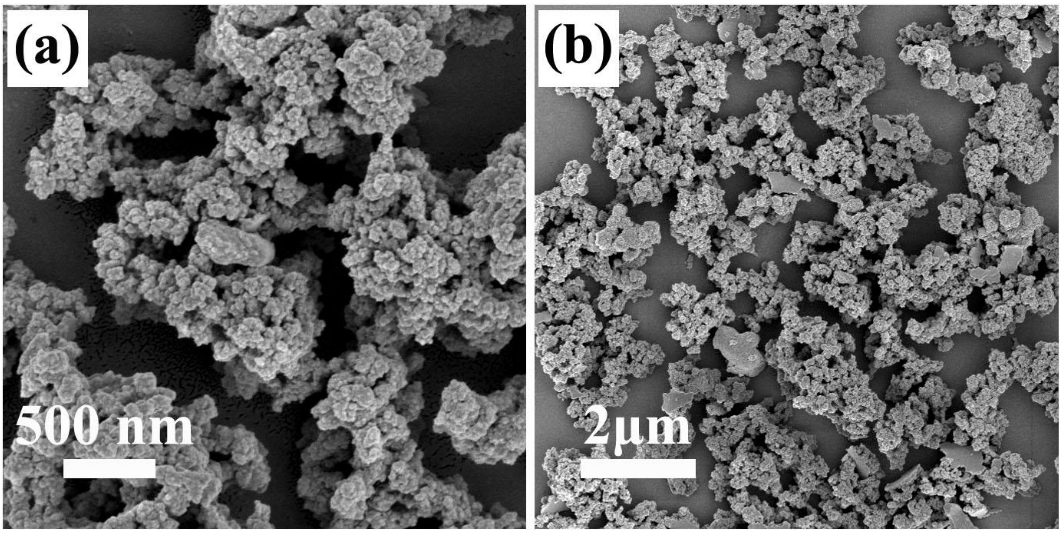

Figure S2. SEM images of ultrasonicated PCCs.

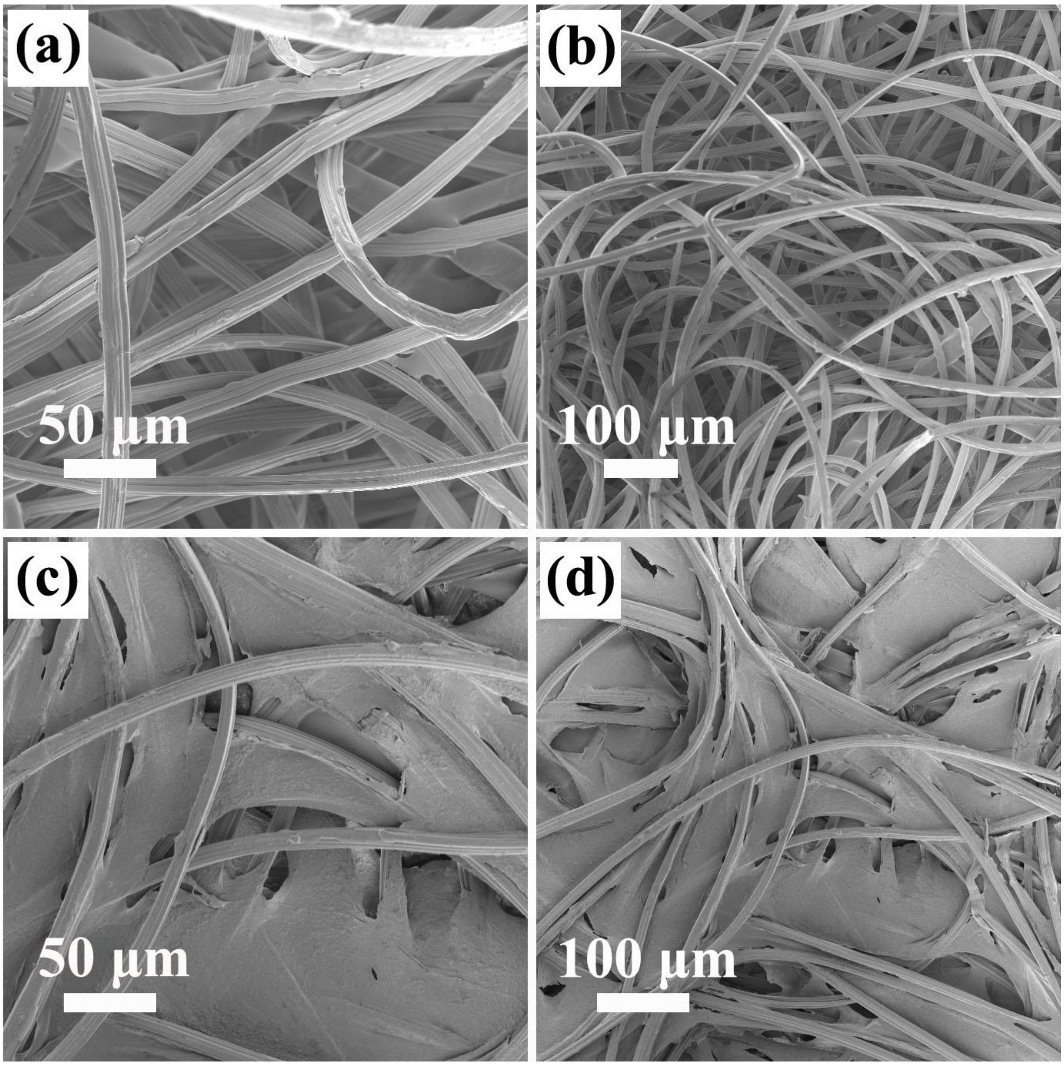

Figure S3. SEM images of original bamboo paper (a, b) and photothermal aerogel sheet (c, d).

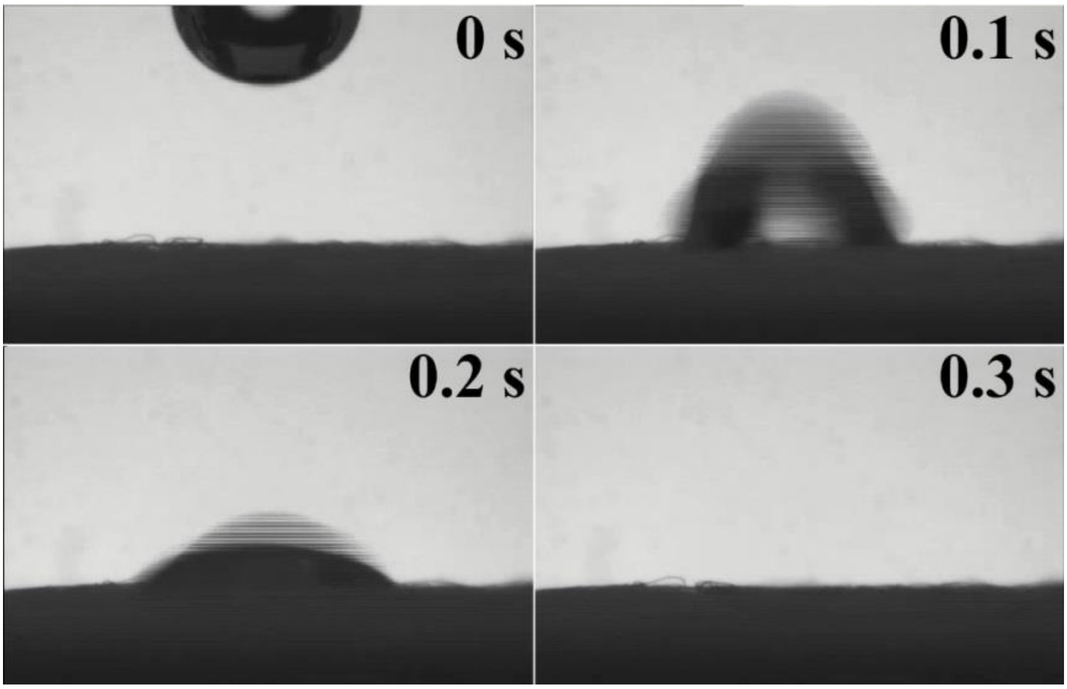

Figure S4. Time-lapse snapshots of absorption of a water droplet by the pure bamboo paper sheet.

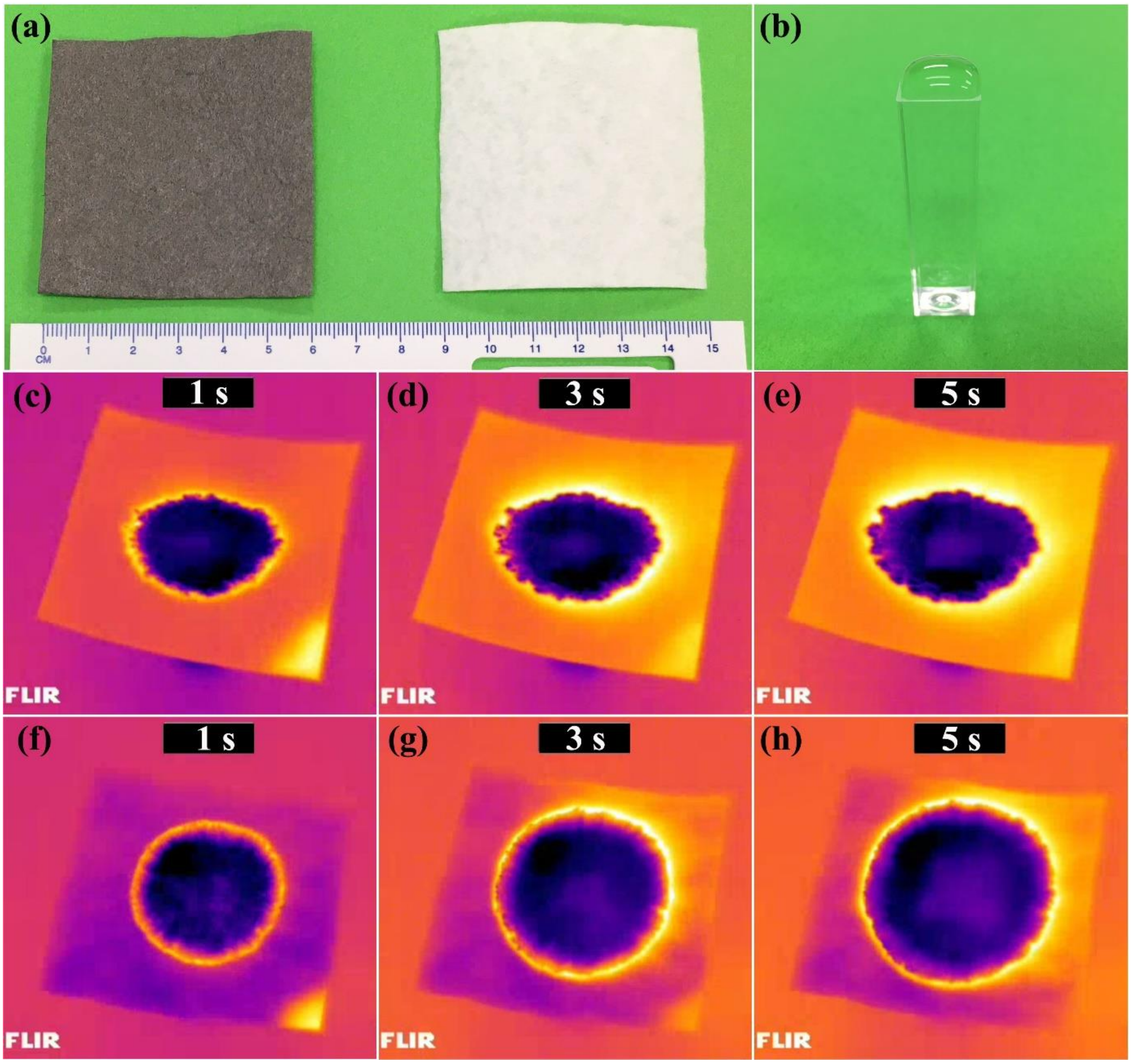

Figure S5. Digital images of the photothermal aerogel sheet (a, left), original bamboo paper (a, right) and cuvette with water over its brim (b) for wicking test. IR images showing the variation of the wicking area of bamboo paper (c-e) and photothermal aerogel sheet (f-h) with different contact time.

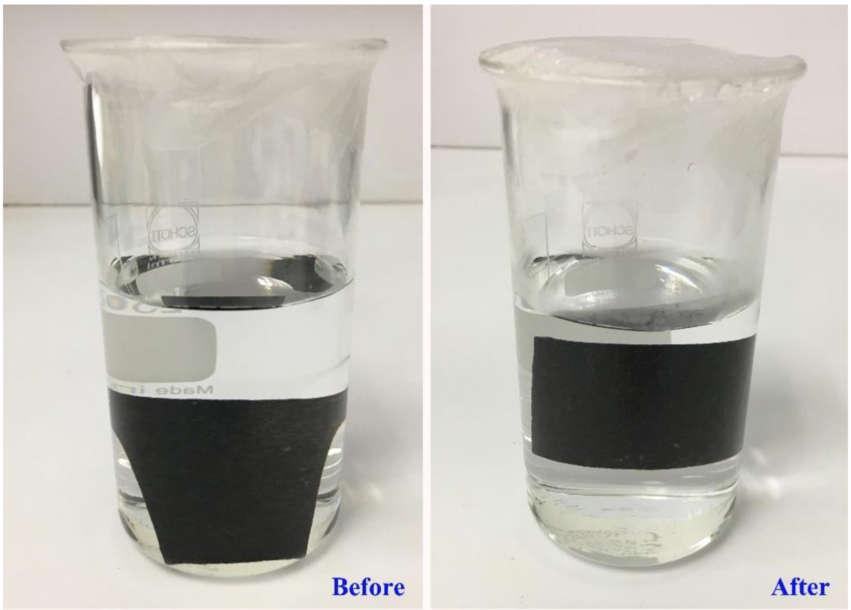

Figure S6. Digital photographs of the photothermal aerogel sheet before and after 8 hours of continuous heating in seawater at 50 °C.

Table S1. Parameter of aerogel sheets for photothermal evaporator construction.

Evaporator series	Fin number / Material	Area of top surface	Fin / side wall parameter
#1 Cylinder	--/PT sheet	26.40 cm^2	18.2 cm (width) × 8 cm (length above water)
#2 Heatsink-like	4/ PT sheet	26.40 cm^2	2 × 5 cm (width) × 8 cm (length above water)<br>2 × 3 cm (width) × 8 cm (length above water)
#3 Heatsink-like	5/ PT sheet	26.40 cm^2	1 × 5.4 cm (width) × 8 cm (length above water)<br>2 × 4.6 cm (width) × 8 cm (length above water)<br>2 × 3 cm (width) × 8 cm (length above water)
#4 Heatsink-like	6/ PT sheet	26.40 cm^2	2× 5.3 cm (width) × 8 cm (length above water)<br>2 × 4.3 cm (width) × 8 cm (length above water)<br>2 × 3 cm (width) × 8 cm (length above water)
#5 Heatsink-like	7/ PT sheet	26.40 cm^2	1× 5.4 cm (width) × 8 cm (length above water)<br>2 × 5 cm (width) × 8 cm (length above water)<br>2 × 4 cm (width) × 8 cm (length above water)
#6 Heatsink-like	6/ pure bamboo paper	26.40 cm^2	2× 5.3 cm (width) × 8 cm (length above water)<br>2 × 4.3 cm (width) × 8 cm (length above water)<br>2 × 3 cm (width) × 8 cm (length above water)

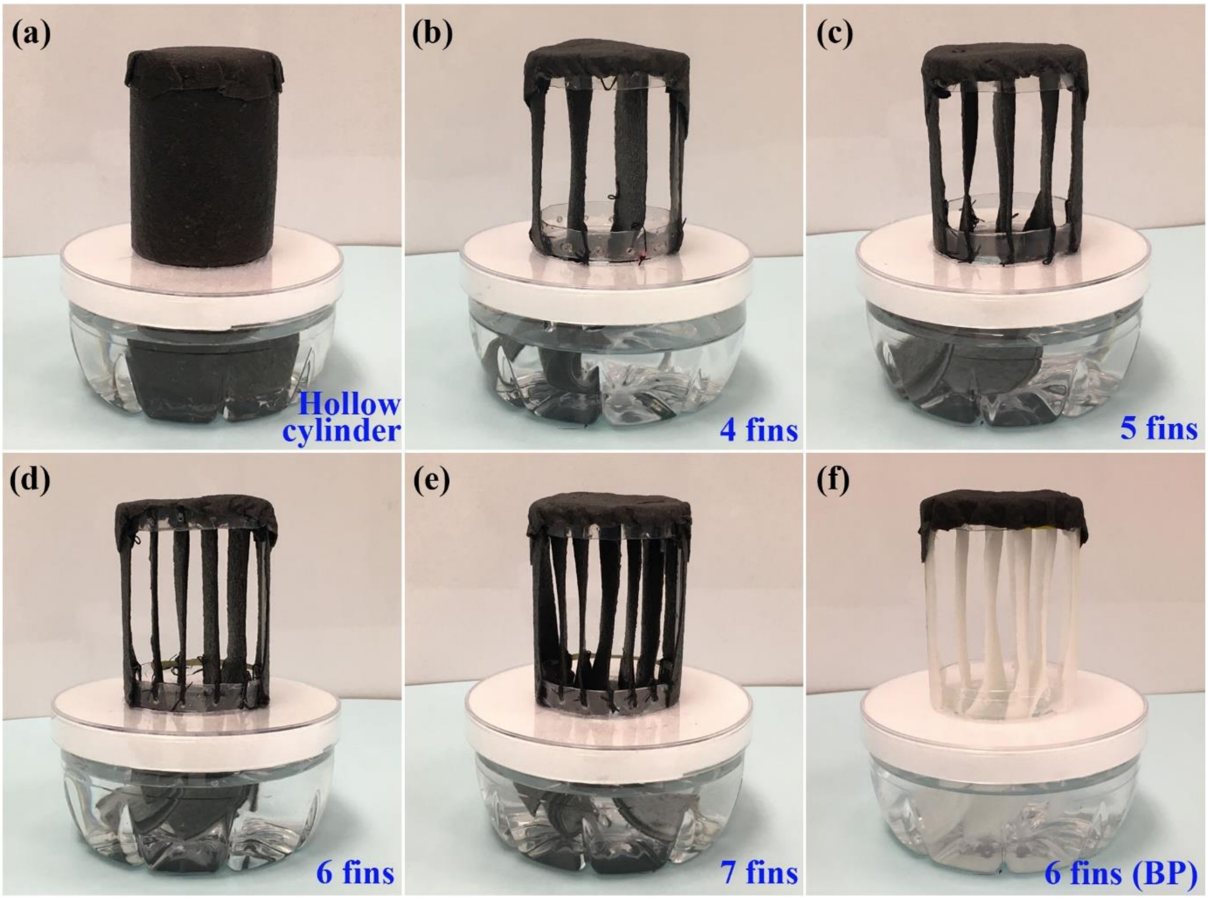

Figure S7. Photograph display of the photothermal evaporator constructed for solar steam generation: hollow cylinder structure (a), 4-fin heatsink-like evaporator (b), 5-fin heatsink-like evaporator (c), 6-fin heatsink-like evaporator (d), 7-fin heatsink-like evaporator (e) and 6-BP fin heatsink-like evaporator (f).

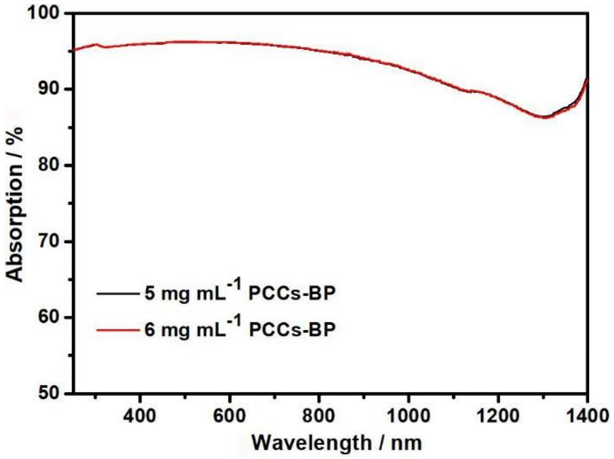

Figure S8. Absorption spectra of the photothermal aerogel sheets (wet) with PCCs concentration of 5 and 6 mg mL^{-1}.

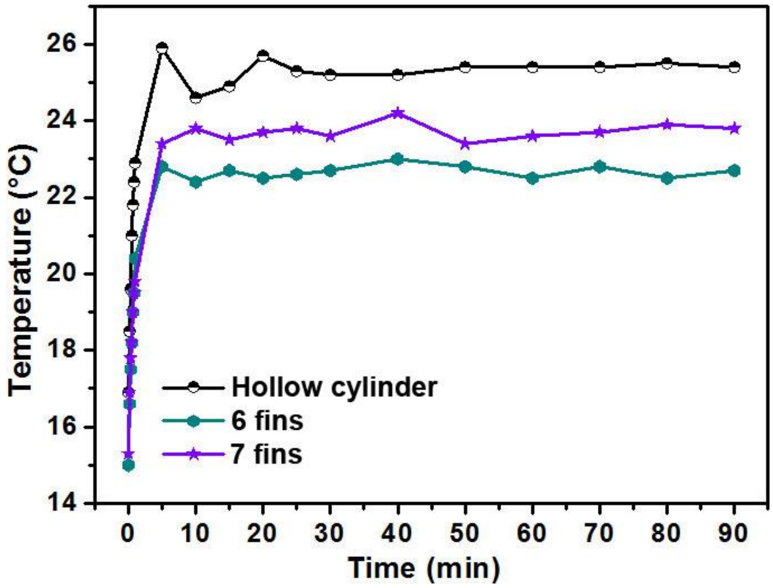

Figure S9. Time-dependent average temperature of the top evaporation surface of different solar steam evaporators.

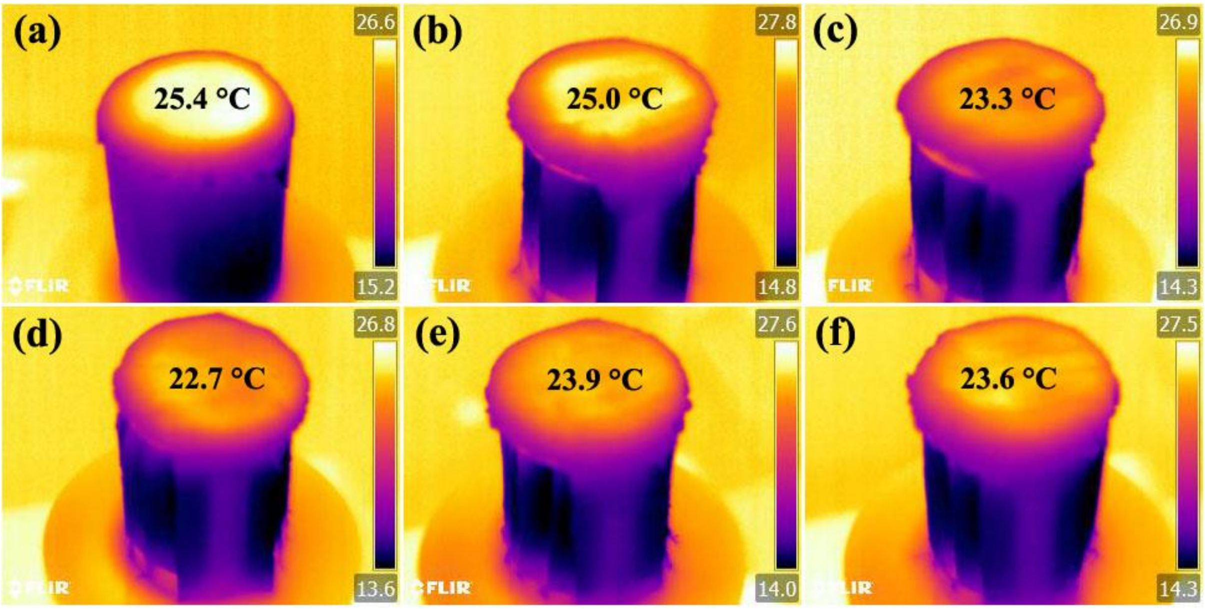

Figure S10. Steady state IR images of one-piece hollow cylinder evaporation structure (a), 4-fin (b), 5-fin (c), 6-fin (d), 7-fin (e) heatsink-like photothermal evaporators and evaporator with 6 bamboo paper fins (f). The value indicates the steady average temperature on the top evaporation surface.

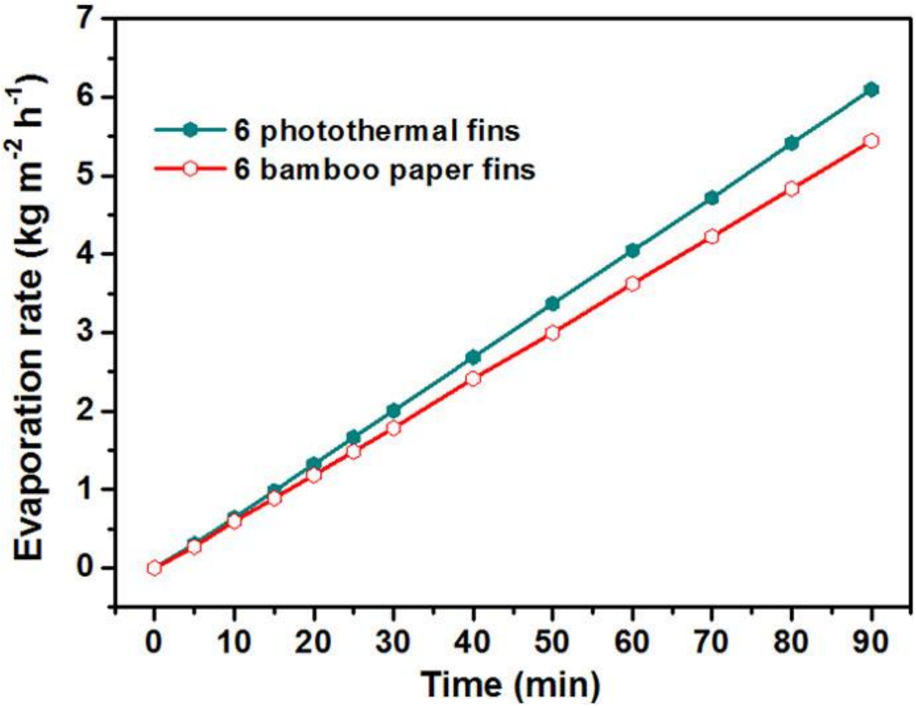

Figure S11. Time-dependent weight loss of water over heatsink-like evaporators with 6 photothermal or pure bamboo paper fins.

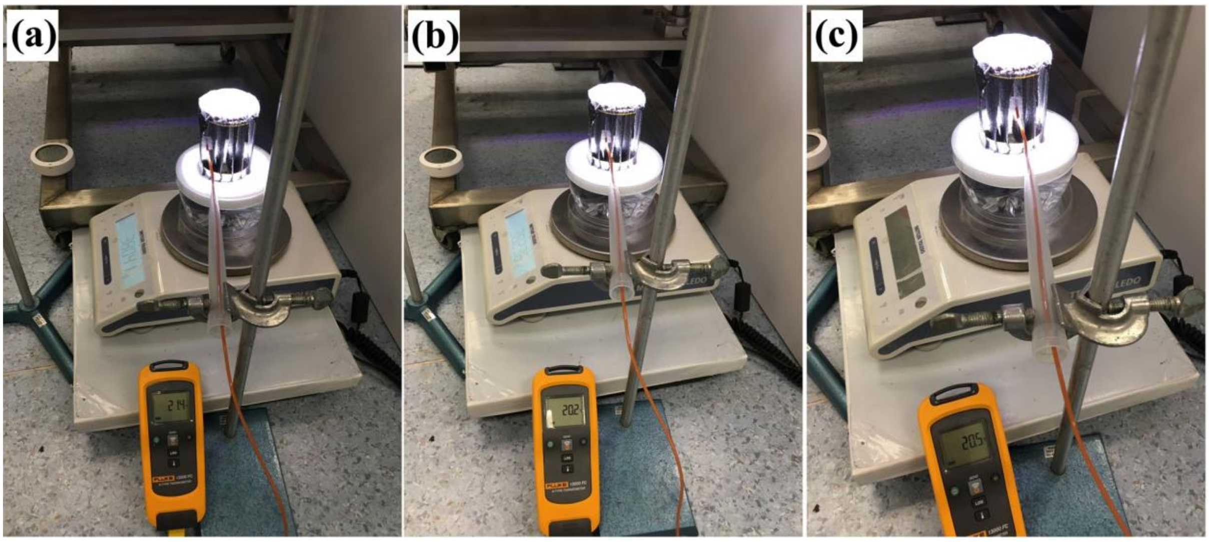

Figure S12. Digital images of thermocouple setup to monitor the temperature of the surrounding medium during evaporation.

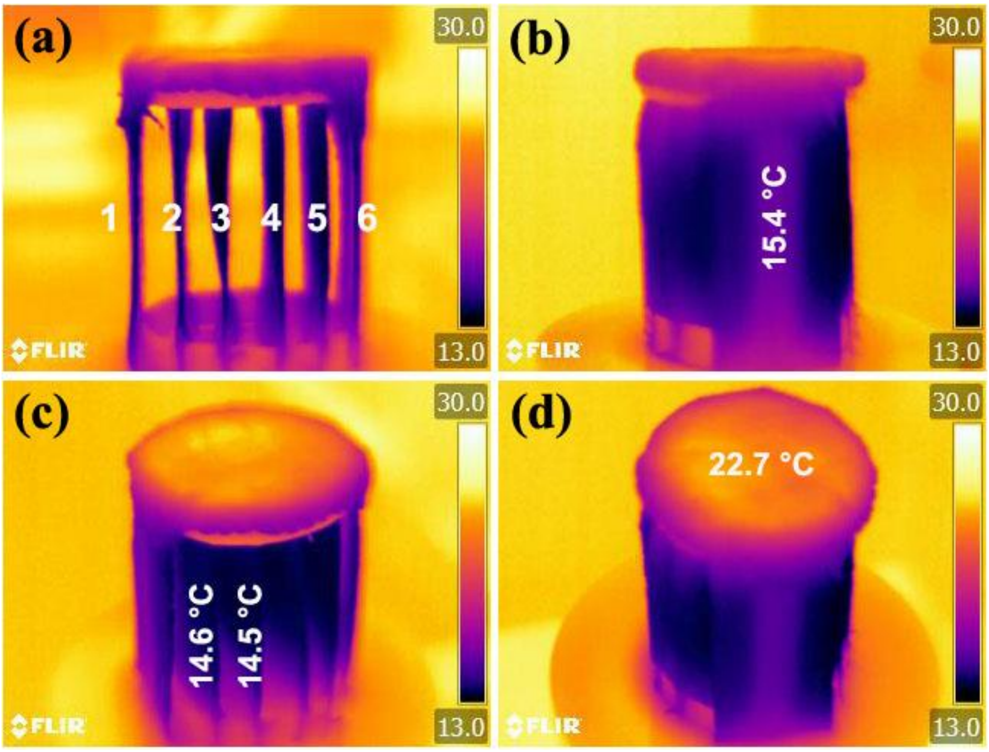

Figure S13. IR images to show the temperature distribution over the 6-fin heatsink-like photothermal evaporator.

Table S2. Energy calculation over the 6 photothermal fin heatsink-like evaporator.

	Light input	Radiation		Convection		Conduction energy exchange with phase water
	αE_{in}	$-A\varepsilon\sigma\,(T^4-T_E^{\ 4})$		$-Ah\,(T-T_E)$		$-cm\Delta T$
Top surface	2.40 W	0.034 W		0.060 W		0.240 W
Fin-1		0.125 W	0.066 W	0.230 W	0.125 W	
Fin-2		0.109 W	0.109 W	0.206 W	0.206 W	
Fin-3		0.136 W	0.136 W	0.258 W	0.258 W	
Fin-4		0.136 W	0.136 W	0.258 W	0.258 W	
Fin-5		0.109 W	0.109 W	0.206 W	0.206 W	
Fin-6		0.066 W	0.125 W	0.125 W	0.230 W	
Total	2.40 W	4.02 W				0.24 W

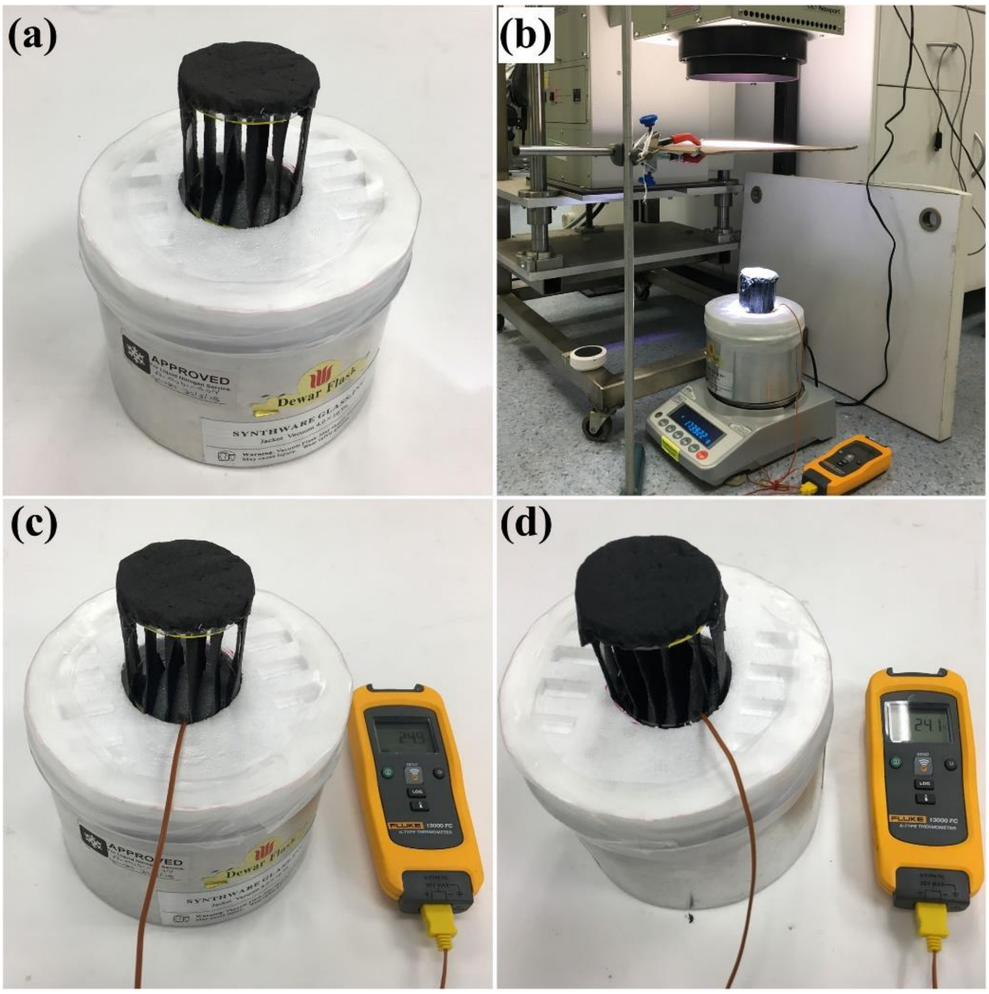

Figure S14. Thermal insulation setup to monitor the temperature variation of bulk water during evaporation under one sun irradiation.

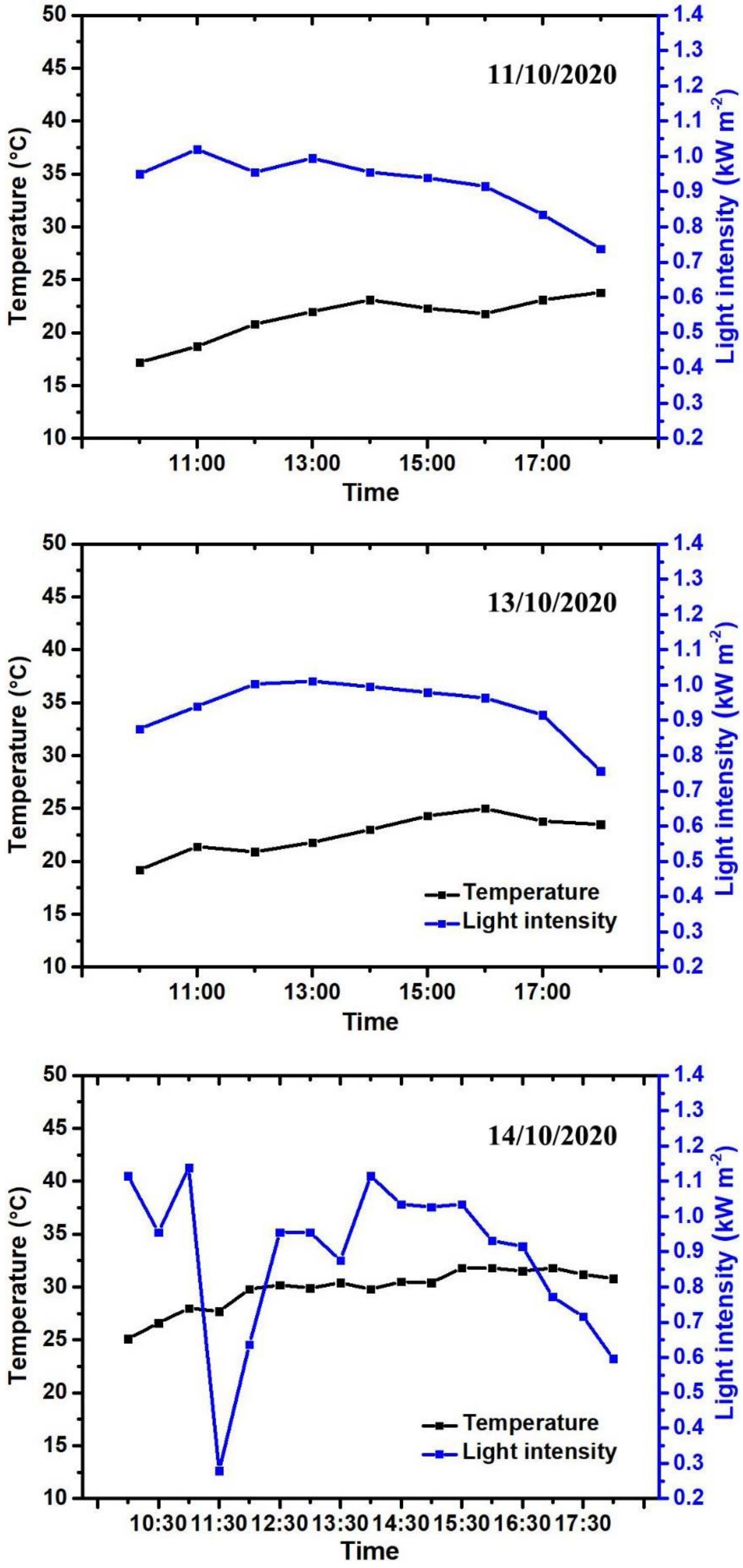

Figure S15. Light intensities and environmental temperatures during the outdoor seawater desalination.

Table S3. Weather conditions and average evaporation rates of the outdoor seawater desalination.

Test date	Weather condition	Evaporated water	Average evaporation rate
11/10/2020	Sunny	57.45 g	2.72 kg m^{-2} h^{-1}
13/10/2020	Sunny	68.57 g	3.24 kg m^{-2} h^{-1}
14/10/2020	Partly Cloudy	59.69 g	2.82 kg m^{-2} h^{-1}

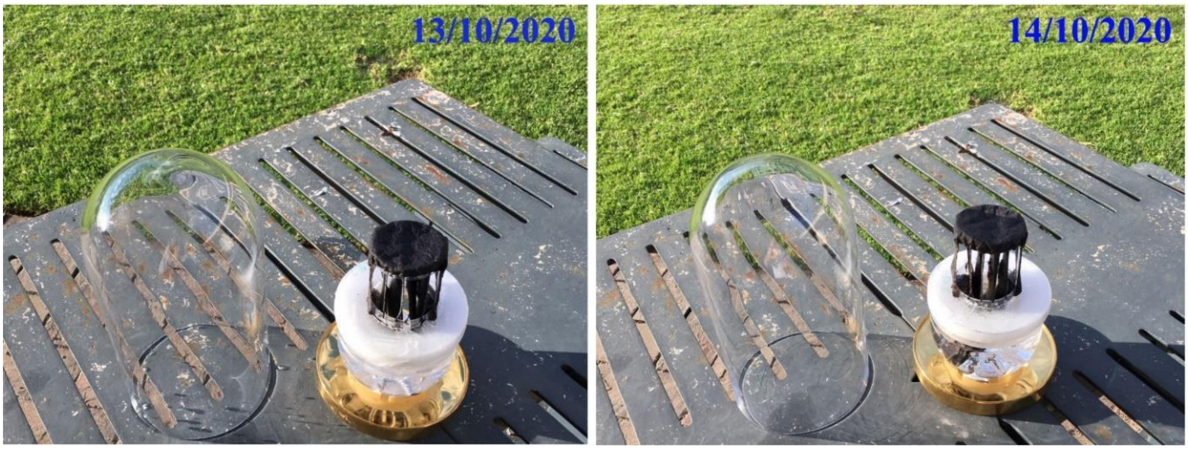

Figure 16. Digital photographs showing no salt accumulation on the surface of the evaporator after 8 hours of continuous seawater evaporation.
